# Celecoxib as a Valuable Adjuvant in Cutaneous Melanoma Treated with Trametinib

**DOI:** 10.3390/ijms22094387

**Published:** 2021-04-22

**Authors:** Diana Valentina Tudor, Ioana Bâldea, Diana Elena Olteanu, Eva Fischer-Fodor, Virag Piroska, Mihai Lupu, Tudor Călinici, Roxana Maria Decea, Gabriela Adriana Filip

**Affiliations:** 1 Department of Physiology, Faculty of Medicine, “Iuliu Hațieganu” University of Medicine and Pharmacy, 400012 Cluj-Napoca, Romania; dianatudor91@gmail.com (D.V.T.); baldeaioana@gmail.com (I.B.); mihailupu5@yahoo.com (M.L.); roxanadecea@yahoo.com (R.M.D.); adrianafilip33@yahoo.com (G.A.F.); 2 “Prof. Dr. Ion Chiricuță” Oncology Institute, 400015 Cluj-Napoca, Romania; fischer.eva@iocn.ro (E.F.-F.); virag.piroska@yahoo.com (V.P.); 3 Department of Medical Informatics and Biostatistics, Faculty of Medicine, “Iuliu Hațieganu” University of Medicine and Pharmacy, 400349 Cluj-Napoca, Romania; tcalinici@umfcluj.ro

**Keywords:** melanoma, COX-2, celecoxib, trametinib, inflammation

## Abstract

Background: Melanoma patients stop responding to targeted therapies mainly due to mitogen activated protein kinase (MAPK) pathway re-activation, phosphoinositide 3 kinase/the mechanistic target of rapamycin (PI3K/mTOR) pathway activation or stromal cell influence. The future of melanoma treatment lies in combinational approaches. To address this, our in vitro study evaluated if lower concentrations of Celecoxib (IC_50_ in nM range) could still preserve the chemopreventive effect on melanoma cells treated with trametinib. Materials and Methods: All experiments were conducted on SK-MEL-28 human melanoma cells and BJ human fibroblasts, used as co-culture. Co-culture cells were subjected to a celecoxib and trametinib drug combination for 72 h. We focused on the evaluation of cell death mechanisms, melanogenesis, angiogenesis, inflammation and resistance pathways. Results: Low-dose celecoxib significantly enhanced the melanoma response to trametinib. The therapeutic combination reduced nuclear transcription factor (NF)–kB (*p* < 0.0001) and caspase-8/caspase-3 activation (*p* < 0.0001), inhibited microphthalmia transcription factor (MITF) and tyrosinase (*p* < 0.05) expression and strongly down-regulated the phosphatidylinositol-3-kinase/protein kinase B (PI3K/AKT) signaling pathway more significantly than the control or trametinib group (*p* < 0.0001). Conclusion: Low concentrations of celecoxib (IC_50_ in nM range) sufficed to exert antineoplastic capabilities and enhanced the therapeutic response of metastatic melanoma treated with trametinib.

## 1. Introduction

Cutaneous melanoma has registered the fastest increasing incidence among malignant tumors [1]. Estimations of its incidence rate showed further increases through 2031 due to aging populations, climate change and rising levels of solar ultraviolet (UV) radiation [2]. UV-induced chronic inflammation orchestrates the interactions of melanoma and immune cells at the tumor milieu, in favor of immune escape and therapeutic resistance [3,4]. This has urged the need for a better biological understanding of the melanoma microenvironment, while also forming the motivation for revolutionizing melanoma treatment in the last decade [5,6]. The gold standard treatment for this condition is surgery, followed by systemic chemo and immunotherapy in more advanced stages [7].

Immunotherapy and targeted therapies have significantly reduced the risk of tumor relapse and increased overall survival in stages III–IV melanoma patients. If the patient has a BRAF–V600E (*B-Raf* proto-oncogene) mutation, treatment with BRAF-inhibitors (dabrafenib, vemurafenib, encorafenib) combined with mitogen activated protein kinase (MEK)-inhibitors (trametinib, cobimetinib, binimetinib) have significantly improved overall survival [8]. However, resistance often occurs after 1 year of BRAF/MEK inhibitor combination because other escape mechanisms evolve [1,9,10,11,12]. Among them, melanoma phenotype plasticity plays a major role [13]. Therefore, new molecules are now being targeted in order to optimize or formulate new therapeutic strategies [14].

The cyclooxygenase-2 (COX-2) enzyme has been proven to represent a negative prognostic marker in numerous cancers, including melanoma [15,16]. The prostaglandin E2 (PGE2)/COX-2 signaling pathway is not only part of melanoma genesis, but may also induce T-cell exhaustion and programmed cell death protein 1/programmed death-ligand 1 (PD-1/PD-L1) overexpression [17,18,19]. Consequently, selective COX-2 inhibitors can be considered as promising cancer chemo-preventive agents. Among them, celecoxib has been proven to be efficient adjuvant in melanoma, despite its incompletely understood antitumor mechanisms [20,21].

Based on these data, our study tested the in vitro use of celecoxib as an adjuvant chemo-preventive agent for trametinib in a simulated human melanoma 2D model. In order to narrow the gap between bench-and-bedside, our study raised the following question: will celecoxib still preserve its antitumor capabilities in lower concentrations (IC_50_ in nM range), enhance the melanoma therapeutic response and block AKT resistance pathway activation? To mimic the real extracellular matrix influence on melanoma cells, experiments were carried out on SK-MEL-28 human melanoma cells and BJ fibroblasts, used as co-culture.

## 2. Materials and Methods

### 2.1. Melanoma Co-Culture Bioassays

#### Cell Co-Culture and Reagents

The SK-MEL-28 human metastatic melanoma cell line (Cell line services, Eppelheim, Germany) was seeded in co-culture with BJ normal fibroblasts (ATCC, Gaithersburg, MA, USA). BJ cell culture was kindly supplied by the “Prof. Dr. Ion Chiricuţă” Oncology Institute of Cluj-Napoca, Romania. SK-MEL-28 melanoma cells were maintained in Roswell Park Memorial Institute (RPMI) 1640 growth medium, while BJ fibroblasts were seeded in Minimum Essential Medium Eagle (MEM). Each medium was enriched with 10% fetal calf serum (FCS), 5 ng/mL amphotericin and 50 μg/mL gentamicin, all from Biochrom AG, Berlin, Germany. Each cell line was used to compare the results of the co-culture. Standard culture conditions were followed: cells were fed twice each week and kept in an incubator at constant temperature (37 °C) and humidity, under a 5% CO_2_ atmosphere; all experiments were conducted in triplicate, and each time cells were used within a maximum of four passages. Trametinib and celecoxib were purchased from Cayman Chemical, MI, USA.

### 2.2. Cytotoxicity Assay

Viability tests were initially performed on each cell line used alone and then on cells used as co-culture. BJ cells were seeded in ELISA 96 wells micro titration flat bottom plates and incubated overnight at 37 °C. After 24 h, SK-MEL-28 cells were added to the plates at a density of 1 × 10^4^/well in order to form the co-culture, and were left to settle for 48 h. Then, co-culture cells were exposed for 24–48–72–96 h either to celecoxib, trametinib or the chosen combination. Experiments started separately with well-established concentrations of celecoxib (ranging from 10, 20, 50, 100, 200, 250 nM) solved in the medium and trametinib (ranging from 2.5, 5, 10, 25, 50, 100 nM) solved in the medium. Two concentrations were chosen for each drug to be further tested in combination: Celecoxib (C1 = 20 nM and C2 = 50 nM) and trametinib (T1 = 25 nM and T2 = 50 nM) solved in the medium. Untreated cells exposed to the medium were used as controls. Following different exposure regimens, viability assays were performed by adding CellTiter 96^®^ AQueous Non-Radioactive Cell Proliferation Assay (Promega Corporation, Madison, WI, USA) directly to wells. After 4 h of incubation, the number of living cells in the co-culture was identified using an ELISA plate reader (Tecan, Mannedorf, Switzerland) that measured the absorbance of formazan (at 540 nm), produced by the metabolically active cells. The results were presented as IC50. Cells were observed using an inverted microscope (Olympus CKX 41, Hamburg, Germany) and pictures were taken using a digital camera (Olympus, E 330, original magnification 10 times).

### 2.3. Experimental Design

In vitro experiments started with normal fibroblasts (BJ) and melanoma cells (SK-MEL-28) seeded in Petri dishes at a density of 1 × 10^4^/cm^2^. After 48 h, the cells were treated with celecoxib, trametinib or the combined regimen, while untreated cells were used as controls. Co-culture cells were further incubated for 72 h and finally tested for cell death induction (Annexin-FITC/PI staining-flow cytometry) and mechanisms involved in cell death and survival: Signaling molecules (nuclear factor-kappa B activation-NF-κB and phosphorylated form pNFκB, protein kinase B activation/inactivation—pan AKT and phosphorylated pan pAKT), melanogenesis (microphthalmia transcription factor—MITF, tyrosinase protein), inflammation (cyclooxygenase-2—COX-2), angiogenesis (vascular endothelial growth factor—VEGF; hypoxia inducible factor–1 alpha—HIF-1α) and stroma remodeling (transforming growth factor beta—TGF-β). Following a well-established protocol, the same method was used for control and treated groups.

**Cell membrane integrity assay.** As a gauge of cell damage, lactate dehydrogenase (LDH) was quantified from the culture medium by colorimetry (Abcam, Cambridge, UK). Data are expressed as nmol NAD^+^/min.

**Cell death mechanisms.** When cell death mechanisms were analyzed, treated co-culture cells were stained with Annexin V-fluorescein isothiocyanate (FITC)/vital dye propidium iodide (PI) (BD Pharmingen Biosciences, San Jose, CA, USA). Viable cells were identified as Annexin V (−)/PI (−), necrotic cells were shown as PI (+), early apoptotic cells were Annexin V-FITC (+), while late apoptosis was Annexin V (+)/PI (+). The separation among these four cell groups was conducted by flow cytometry, using a BD FACS Canto II flow cytometer (Becton Dickinson & Company, Franklin Lakes, NJ, USA). The flow cytometric detection was completed using two lasers as excitation sources: Red (633 nm, 17 mWHeNe) and blue (488 nm, air cooled, 20 mW solid state), as previously described.

**Apoptosis.** ELISA analysis of caspase-8 and caspase-3 (CPP32) (Immunoassay kits from R&D Systems, Minneapolis, MN, USA) was performed in order to evaluate the apoptosis mechanisms. After exposing co-culture cells to the established therapy, cells supernatants were treated according to manufacturer’s instructions for caspase-3 and caspase-8. Readings were performed at 450 nm using a correction wavelength set at 540 nm, utilizing ELISA plate reader (Tecan).

**Inflammation, melanogenesis and cell signaling pathways assessment.** The signaling pathways involved in melanoma cell death and survival were assessed via Western blot (WB) analysis. Following the different exposure regimens, cells were incubated for 72 h, collected and prepared for cell lysates following the Bradford method (Biorad, Hercules, CA, USA), setting bovine serum albumin as the standard. All lysates obtained were corrected by total protein concentration. Afterwards, cell lysates (20 μg protein/lane) were separated via electrophoresis on SDS PAGE gels and further transferred to polyvinylidenedifluoride membranes, utilizing the Biorad Miniprotean system (BioRad, Hercules, CA, USA). Protein migration was blocked, the WB membranes were subsequently incubated overnight at 4 °C with primary antibodies against: NF-κB (1:1000), phospho-NF-κB (1:1000) p65 (Ser536) (93H1) (pNF-kB), pan AKT (1:500), phospho-panAKT (1:500) (pan pAKT), COX-2 (1:500), HIF-1α (1:500), tyrosinase (1:1000), MITF (1:500) (Santa Cruz Biotechnology, Delaware Ave, Santa Cruz, CA, USA), TGF-β (1:1000) (Abcam plc., Cambridge, UK) and GAPDH (1:1000), then washed and further incubated for 1 h, at room temperature with corresponding secondary antibodies (Santa Cruz Biotechnology, Delaware Ave, Santa Cruz, CA, USA). Supersignal West Femto Chemiluminiscent substrate (Thermo Fisher Scientific, Rockford, IL, USA) and Gel Doc Imaging system equipped with a XRS camera and Quantity One analysis software (Biorad, Hercules, CA, USA) were used to detect the proteins of interest. The protein loading control was GAPDH (Trevigen Biotechnology Gaithersburg, MD, USA). The volume for each WB lane was measured using Image Lab Bio-Rad software.

**Angiogenesis.** VEGF expression was assessed by ELISA and HIF-1α by WB as key mediators of angiogenesis in co-culture cells. For VEGF evaluation ELISA Immunoassay kit from R&D Systems, Inc. (Minneapolis, MN, USA) was used. Co-culture cell supernatants were treated according to the manufacturer’s specifications. An ELISA plate reader (Tecan) performed readings at 450 nm with correction wavelength set at 540 nm. HIF-1 alpha (Santa Cruz Biotechnology, Delaware Ave, Santa Cruz, CA, USA) was assessed by WB as previously described.

**AKT pathway activation.** AKT activation was assessed by WB using an AKT inhibitor-iAKT (Cell Signaling Technology, Leiden, The Netherlands) for 24 h on a separate group of co-culture cells. Cells were subsequently washed, treated with celecoxib and trametinib combination according to the mentioned protocol. Lysates were used for WB testing in order to evaluate AKT pathway activation (pan AKT, pan pAKT, i AKT and i pAKT) in the group of interest.

**Stroma remodeling.** TGF-β was evaluated as a marker for stroma remodeling and tumor growth. In our melanoma experimental model TGF-β (Abcam plc., Cambridge, UK) expression was assessed by WB.

**Statistical method**. All data were collected using Microsoft Excel for Windows 10 and then analyzed using the one-way ANOVA, Student t test, Tukey Posttests and Nonlinear regression (curve fit) via statistical package Prism version 8.00 for Windows, GraphPad Software, San Diego, CA, USA. The difference between control and treated group was considered statistically significant when *p* value was less than 0.05. In order to establish the most suitable drug dose for the therapeutic combination, the inhibitory concentration 50% (IC_50_) was calculated using nonlinear regression (curve fit) dose–response-Inhibition. The panel illustrating the drugs combination was created using Excel for Microsoft 365.

## 3. Results

### 3.1. Cell Viability Assay

**Figure 1:**
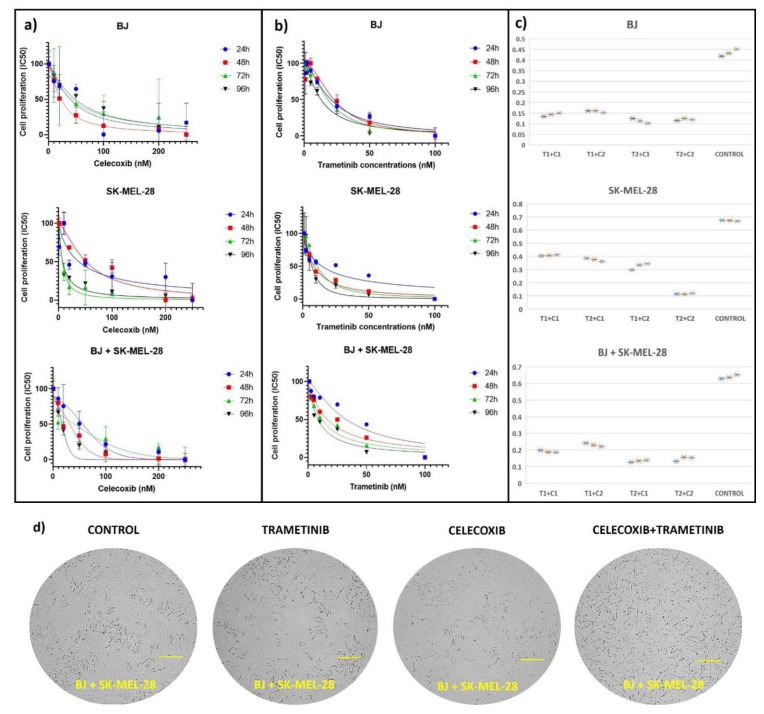
Cell proliferation testing after exposure to celecoxib and trametinib. Each cell line, SK-MEL-28 and BJ cell cultures, as well as the co-cultured cells were exposed to different concentrations of celecoxib (for 24, 48, 72, 96 h) (**a**), trametinib (for 24, 48, 72, 96 h) (**b**) and to the celecoxib (C1 = 20 nM and C2 = 50 nM) and trametinib (T1 = 25 nM and T2 = 50 nM) drug combination for 72 h (**c**). Both (**a**,**b**) IC50 graphs were generated using GraphPad Software, nonlinear regression (curve fit) and illustrate mean values ± standard error of the mean (SEM), *n* = 3 for each sample. The third panel from both (**a**,**b**) graphs illustrates cell viability results after treating co-culture cells with celecoxib or trametinib separately. The suitable drug combination was chosen after analyzing cell viability after 72 h exposure to four different drug combinations via Excel–Box and Whisker. Each drug combination was testes in triplicate and was illustrated as asterix (*) after Tecan reading (**c**). Cell viability of the BJ + SK-MEL-28 co-culture diminished with increasing concentrations of celecoxib and trametinib, in a dose and time dependent manner. At the end of the protocol, following different exposure regiments, cells were observed in each group as shown in (**d**).

In order to establish the most suitable doses of celecoxib (C) and trametinib (T) drug combination, viability tests were initially performed on SK-Mel-28 and BJ co-cultured cells compared to each cell line used alone. Viability quantified by colorimetry suggested a dose and time dependent effect. Initially, each drug was used in six different concentrations for four different exposure times (24 h, 48 h, 72 h, 96 h), as follows: celecoxib 10, 20, 50, 100, 200, 250 nM (Figure 1a) and trametinib 2.5, 5, 10, 25, 50, 100 nM (Figure 1b). Next, the best two IC50 concentrations were chosen for both celecoxib (C1 = 20 nM and C2 = 50 nM) and trametinib (T1 = 25 nM and T2 = 50 nM) to be tested as a combination (T + C) for 72 h on BJ, SK-MEL-28 and co-culture (Figure 1c). Finally, the time exposure was set at 72 h for both celecoxib (LogIC_50_ = 1.267 nM, IC_50_ = 18.48 nM, R^2^ = 0.8296) and trametinib (Log IC_50_ = 1.197 nM, IC_50_ = 15.73 nM, R^2^ = 0.9352) in BJ + SK-MEL-28 co-culture cells. Accordingly, all experiments were performed using 50 nM celecoxib and 25 nM trametinib for further measurements.

### 3.2. Cell Membrane Integrity Assay

**Figure 2:**
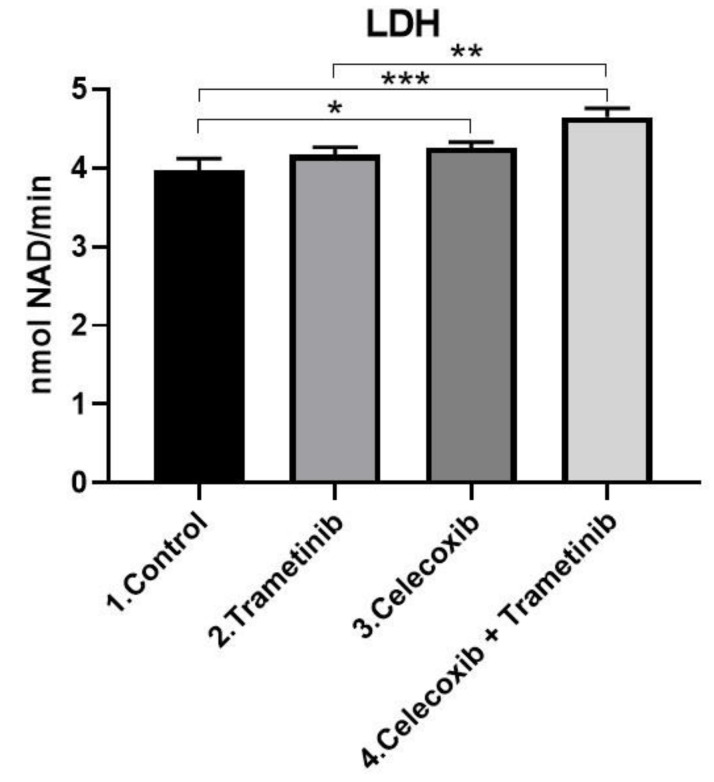
Cell membrane damage. Comparative analysis following T + C exposure compared with the control showed greater cytosolic LDH enzyme release in the last group (*p* < 0.001). LDH levels were even significantly higher than the group treated with celecoxib (*p* < 0.01). Quantitative results were expressed in nmol NAD^+^/min. * *p* < 0.05, *** *p* < 0.001 vs. control, untreated cells; ** *p* < 0.01 vs. trametinib. Each bar represents mean ± standard deviation (*n* = 3).

In the 2D co-culture LDH enzyme levels in media significantly increased in the T + C group after 72 h exposure (mean difference 0.6780, 95% CI 0.3907 to 0.9652, *p* < 0.001) compared with the control, as shown in Figure 2. Where cell membranes are damaged as a result of the cytotoxic effect of the drugs used, LDH enzyme pass into the extracellular space. The LDH levels increased gradually compared to the control, in the last group being even higher than celecoxib or trametinib used alone (mean difference 0.4831, 95% CI 0.1959 to 0.7703, *p* < 0.01). This suggests a cumulative effect of T + C in regard to cell membrane damage and cell death. LDH release in the trametinib group compared to the control was not significant (*p* = 0.2104 > 0.05).

### 3.3. Cell Death Mechanism

**Figure 3:**
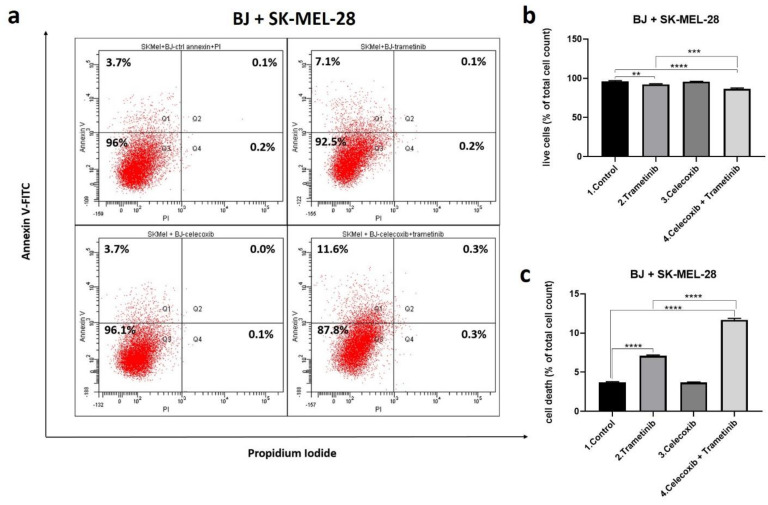
Cell death assessment after 72 h. Comparative FACS analysis following T + C treatment versus control in SK-MEL-28 and BJ fibroblasts co-culture cells; quantitative FACS results for BJ + SK-MEL-28 are expressed as % of total cell count-annexin V and PI positive cells, from the total cell number (**a**); viable cells (**b**) as well as early and late apoptosis (**c**) were statistically analyzed. Cell death induced by celecoxib group was not significant. Celecoxib used alone did not alter cell viability, while celecoxib added to trametinib enhanced apoptosis mediated cell death in the last group compared to control and trametinib group. ** *p* < 0.01, **** *p* < 0.0001 vs. control, untreated cells and *** *p* < 0.001, **** *p* < 0.0001 vs. trametinib group. Each bar represents mean ± standard deviation (*n* = 3).

Flow cytometry (fluorescence activated cell sorting-FACS) was used to assess cell death mechanisms using annexin/PI staining of treated cells after 72 h exposure to each treatment regimen (Figure 3a–c). The percentages of apoptosis-related dead cells (Q1 + Q2) compared to viable cells (Q3) were analyzed (b and c). In BJ + SK-MEL-28 co-culture celecoxib (50 nM) used alone showed no significant cell death induction compared to the control, correlating with viability tests. Despite this, the percentage of dead cells obtained in the T + C group was significantly increased compared to the control (mean difference 7.967, 95%CI 7.621 to 8.313, *p* < 0.0001) and the trametinib group (mean difference 4.567, 95% CI 4.913 to 4.221, *p* < 0.0001). The main cell death mechanism was apoptosis for the last group treated with the combined therapy. T + C combination potentiated the antitumor effect in the co-culture. These results showed that celecoxib effectively increased trametinib induced cell death via apoptosis. This effect might lead to a better therapeutic response overcoming the melanoma activation of survival mechanisms.

**Figure 4:**
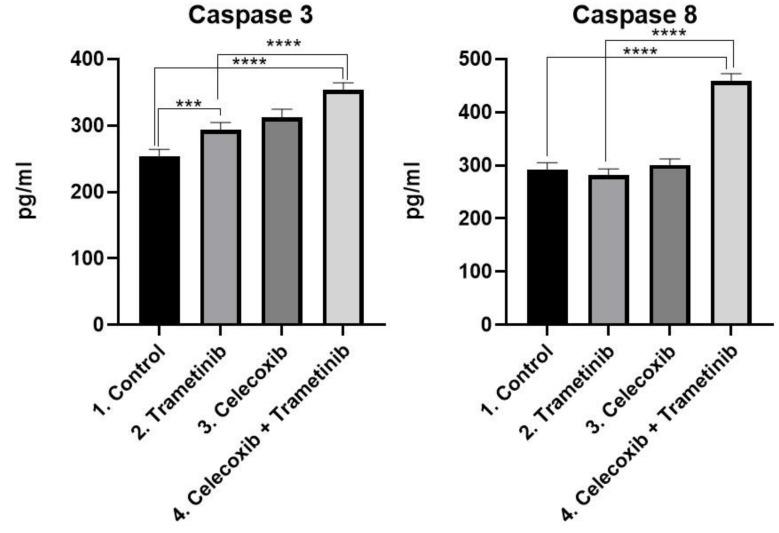
ELISA analysis of caspase-3 and caspase-8 activation as markers for apoptosis. Protein levels of caspase-8 and caspase-3 (pg/mL) in BJ + SK-MEL-28 co-culture cells treated with T + C therapeutic combination for 72 h. *** *p* < 0.001, **** *p* < 0.0001 vs. control or untreated cells and **** *p* < 0.0001 vs. trametinib group. Each bar represents mean ± standard deviation (*n* = 3).

**Apoptosis.** Consistent with the cell death induction obtained by FACS, the ELISA assay showed that melanoma cells underwent apoptosis via caspase-8/caspase-3 activation, after T + C treatment (Figure 4). The activation of caspase-8 (mean difference166.3, 95% CI 143.8 to 188.8, *p* < 0.0001) and caspase-3 (mean difference 99.14, 95%CI 78.87 to 119.4, *p* < 0.0001) was significantly higher in the T + C group compared to untreated cells. The therapeutic combination registered higher caspase activation compared to the trametinib group as well: Caspase-8 (mean difference 176.9, 95% CI 154.4 to 199.4, *p* < 0.0001) activation and caspase-3 (mean difference 60.0, 95% CI 39.72 to 80.27, *p* < 0.0001) activation.

### 3.4. Inflammation, Melanogenesis, Angiogenesis and Signaling Molecules Assessment

**Figure 5:**
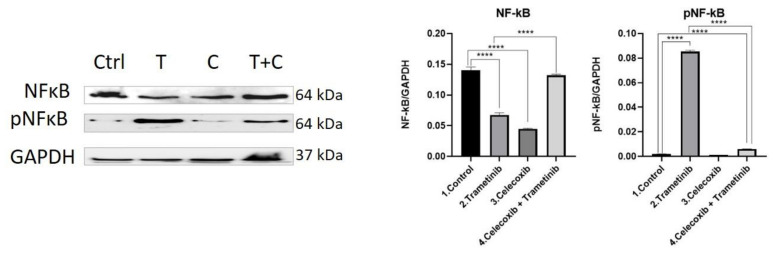
Western blot analysis of NF-kB and pNF-kB. Protein expression of NF-kB and pNF-kB in BJ+ SK-MEL-28 co-culture treated with trametinib (25 nM) and celecoxib (50 nM). WB bands were analyzed by densitometry and results were normalized to GAPDH. The left panel illustrates WB bands (Ctrl = control, T = trametinib, C = celecoxib, T + C = trametinib + celecoxib), while right panels illustrate the quantitative analysis of WB results (1 = control, 2 = trametinib, 3 = celecoxib, 4 = celecoxib + trametinib). **** *p* < 0.0001 vs. control cells; **** *p* < 0.0001 vs. trametinib group. Each bar represents mean ± standard deviation (*n* = 3).

**NF-κB activation.** Melanoma cells often activate the NF-κB pathway in order to achieve survival, proliferation and resistance to apoptosis. Following different exposure regiments, BJ + SK-MEL-28 co-cultured cells treated with celecoxib (C) and trametinib (T) were evaluated for protein expression of the total and the active form of NF-κB, using WB (Figure 5). Even though Trametinib induced the highest level of active protein, the total amount of NF-κB was significantly reduced compared to the control group (mean difference 0.0727, 95% CI 0.0638 to 0.08168, *p* < 0.0001), as shown in Figure 4. Moreover, the last group (T + C) reduced neither the expression of total NF-κB protein (not significant), nor the active form (mean difference 0.00398, 95% CI 0.002623 to 0.005338, *p* < 0.0001) compared to the control. There was also a significant increase in NF-κB protein expression induced by T + C compared to T group (mean difference 0.064744, 95% CI 0.05579 to 0.07368, *p* < 0.0001). However, only a modest part of the NF-κB protein was represented by the active form (Mean difference 0.07935, 95% CI 0.07799 to 0.08070, *p* < 0.0001). Even though celecoxib addition to trametinib significantly increased the total amount of NF-κB expression, the phosphorylated protein was significantly lower compared to trametinib (*p* < 0.0001). This shows that even though the total amount of NF-κB protein was increased following the T + C regimen, the activation pathway was still strongly inhibited in the end by the therapeutic option, suggesting a more potent cell death induction (Figure 5).

**Figure 6:**
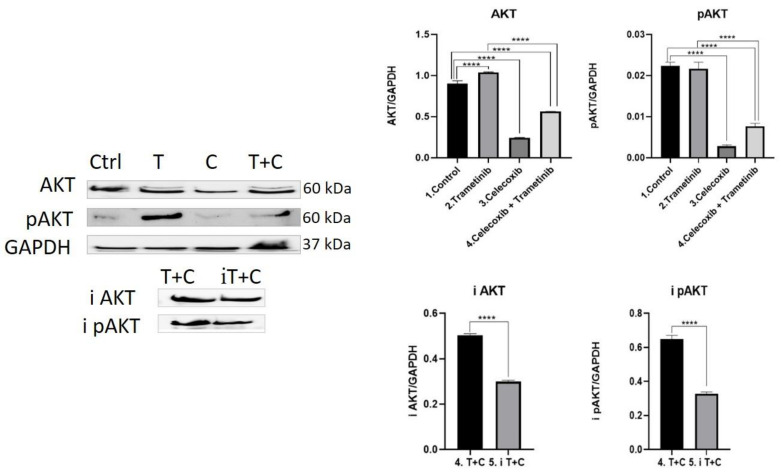
Western blot analysis of AKT pathway activation. Protein expression pan AKT, pan pAKT, i AKT and i pAKT in co-culture cells treated with trametinib (25 nM) and celecoxib (50 nM). WB bands were analyzed by densitometry and results were normalized to GAPDH. The left panel illustrates WB bands (Ctrl = control, T = trametinib, C = celecoxib, T + C = trametinib + celecoxib), while right panels illustrate the quantitative analysis of WB results (1 = control, 2 = trametinib, 3 = celecoxib, 4 = celecoxib + trametinib, 5 = AKT pathway inhibitor + celecoxib + trametinib). **** *p* < 0.0001. Each bar represents mean ± standard deviation (*n* = 3).

**AKT activation.** The PI3K/AKT pathway (phosphatidylinositol 3-kinase) is one of the most important signaling networks in melanoma, frequently used by the tumor cells as an alternative pathway to RAS-RAF-MEK-ERK activation. Therefore, the effect of the therapeutic combination on AKT pathway activation was assessed. As shown in Figure 6, cells treated with trametinib registered a higher level of expression of AKT compared to controls (mean difference 0.1349, 95% CI 0.09001 to 0.1797, *p* < 0.0001). Nevertheless, AKT activation in the trametinib group compared to control was not significant. When cells were treated with the T + C combination, both pan AKT (mean difference 0.3407, 95% CI 0.2959 to 0.3856, *p* < 0.0001) and pan pAKT (mean difference 0.01472, 95% CI 0.01211 to 0.01732, *p* < 0.0001) levels were significantly reduced compared to the control and trametinib group (*p* < 0.0001), suggesting important inhibition of cell survival and proliferation. Celecoxib induced the strongest inhibitory effect on pan AKT and pan pAKT (mean difference 0.01955, 95%CI 0.01694 to 0.02216, *p* < 0.0001), compared to controls the effect aiding to trametinib in the combination therapy. Next, an AKT pathway inhibitor was tested for 24 h on the group of interest (T + C). Both WB bands and quantitative analysis for AKT (i AKT) and pAKT (i pAKT) showed a significant reduction in AKT activation to almost half (*p* < 0.0001).

**Tyrosinase.** Aggressive melanocytic tumors, such as melanoma, overexpress the pigmentation enzyme tyrosinase during tumorigenesis. Tyrosinase activity was measured as a marker of melanogenesis, proving to be significantly inhibited in the group treated with the T + C combination compared to the control (mean difference 0.02466, 95% CI 0.01013 to 0.03919, *p* < 0.01) and the trametinib group (mean difference 0.01576, 95% CI 0.001226 to 0.03029, *p* < 0.05). The enzyme inhibition expressed by trametinib alone compared with the control group did not prove to be statistically significant. Celecoxib diminished tyrosinase activity more than in the control group (mean difference 0.03521, 95% CI 0.02068 to 0.04974, *p* < 0.001) and enhanced the anti-melanogenic effect in the T + C group.

**Figure 7:**
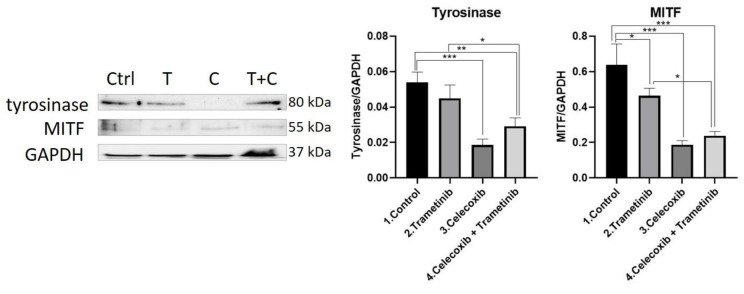
Western blot analysis of tyrosinase and MITF. Protein expression of tyrosinase and MITF in the 2D melanoma model. WB bands were analyzed by densitometry and results were normalized to GAPDH. The left panel illustrates WB bands (Ctrl = control, T = trametinib, C = celecoxib, T + C = trametinib + celecoxib), while right panels illustrate the quantitative analysis of WB results (1 = control, 2 = trametinib, 3 = celecoxib, 4 = celecoxib + trametinib). * *p* < 0.05, ** *p* < 0.01, *** *p* < 0.001. Each bar represents mean ± standard deviation (*n* = 3).

**MITF.** MITF protein levels were measured for melanogenesis assessment in treated BJ + SK-MEL-28 co-culture cells. The T + C therapeutic combination managed to inhibit MITF expression more than on the untreated cells (mean difference 0.4012, 95%CI 0.2313 to 0.5711, *p* < 0.001) and trametinib group (mean difference 0.2263, 95% CI 0.05638 to 0.3962, *p* < 0.05), indicating a potent anti-melanogenic effect. Celecoxib alone had the strongest inhibitory effect on MITF levels compared to the control (mean difference 0.4515, 95% CI 0.2816 to 0.6214, *p* < 001), adding the anti-pigmentation effect to the therapeutic combination. WB results for tyrosinase and MITF protein expression are shown in Figure 7.

**Figure 8:**
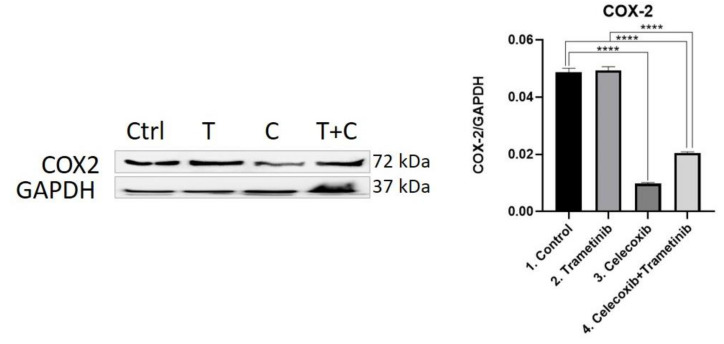
Western blot analysis of COX-2. Protein expression of COX-2 in BJ + SK-MEL-28 co-culture cells. WB bands were analyzed by densitometry and results were normalized to GAPDH. The left panel illustrates WB bands (Ctrl = control, T = trametinib, C = celecoxib, T + C = trametinib + celecoxib), while right panels illustrate the quantitative analysis of WB results (1 = control, 2 = trametinib, 3 = celecoxib, 4 = celecoxib + trametinib). **** *p* < 0.0001. Each bar represents mean ± standard deviation (*n* = 3).

**COX-2.** The COX-2 enzyme was considered a valuable prognostic factor and therapeutic target in melanoma. Thus, COX-2 was evaluated as an inflammation marker via WB. The therapeutic combination of T + C strongly inhibited COX-2 expression compared to the control group (mean difference 0.02827, 95% CI 0.02574 to 0.03080, *p* < 0.0001) and trametinib used alone (mean difference 0.02884, 95% CI 0.02631 to 0.03137, *p* < 0.0001), managing to reduce tumor-associated inflammation. As expected, celecoxib had the strongest inhibitory effect on COX-2 levels compared to controls (mean difference 0.03901, 95% CI 0.03649 to 0.04154, *p* < 0.0001), while trametinib showed no significant inhibitory activity (Figure 8).

**Figure 9:**
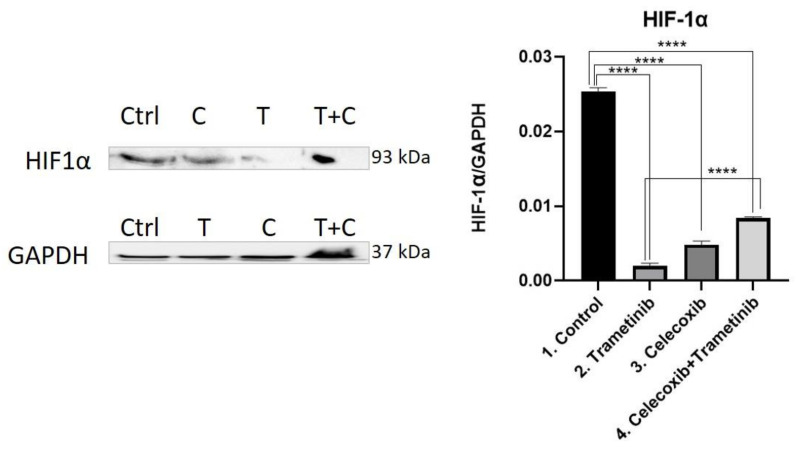
Western blot analysis of HIF-1α. Protein expression of HIF-1α in BJ + SK-MEL-28 co-culture cells. WB bands were analyzed by densitometry and results were normalized to GAPDH. The left panel illustrates WB bands (Ctrl = control, T = trametinib, C = celecoxib, T + C = trametinib + celecoxib), while right panels illustrate the quantitative analysis of WB results (1 = control, 2 = trametinib, 3 = celecoxib, 4 = celecoxib + trametinib). **** *p* < 0.0001. Each bar represents mean ± standard deviation (*n* = 3).

**HIF-1α.** The therapeutic combination of T + C showed an important inhibition of angiogenesis after reducing HIF-1α levels to a greater degree than in the control group (mean difference 0.01691, 95% CI 0.01584 to 0.01799, *p* < 0.0001), but proved to be less efficient than the trametinib group (Mean difference 0.0064, 95% CI 0.005327 to 0.007474, *p* < 0.0001). Trametinib used alone on BJ + SK-MEL-28 co-culture cells showed the greatest inhibitory effect on HIF-1α compared to the control group (mean difference 0.02331, 95% CI 0.02224 to 0.02439, *p* < 0.0001), celecoxib (mean difference 0.002853, 95% CI 0.001780 to 0.003927, *p* < 0.001) and the last group of interest (Figure 9).

**Figure 10:**
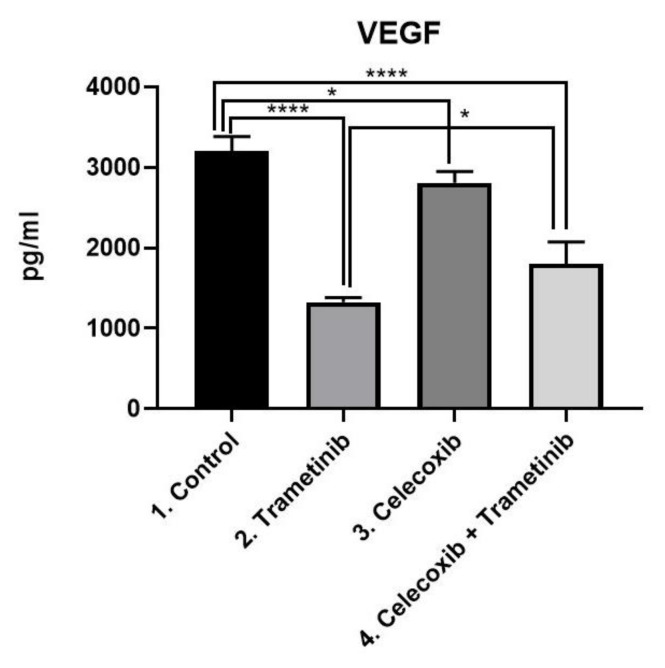
ELISA analysis of VEGF. Protein expression of VEGF (pg/mL) in SK-MEL-28 + BJ co-culture cells treated with T + C therapeutic combination for 72 h. * *p* < 0.05, **** *p* < 0.0001 vs. control, untreated cells and * *p* < 0.05 vs. trametinib group. Each bar represents mean ± standard deviation (*n* = 3).

**VEGF.** In BJ + SK-MEL-28 co-culture cells VEGF was evaluated as a marker of neoangiogenesis via ELISA (Figure 10). The T + C group managed to reduce VEGF expression more than the control (mean difference 1401, 95% CI 1025 to 1778, *p* < 0.0001) and celecoxib group (mean difference 1009, 95% CI 631.8 to 1385, *p* < 0.0001). Trametinib used alone showed the greatest antiangiogenic effect among the four groups, with significantly lower VEGF levels than the control (mean difference 1879, 95% CI 1502 to 2255, *p* < 0.001), celecoxib alone (mean difference 1486, 95% CI 1109 to 1863, *p* < 0.0001) and the last group (mean difference 477.5, 95% CI 100.8 to 854.2, *p* < 0.05).

**Figure 11:**
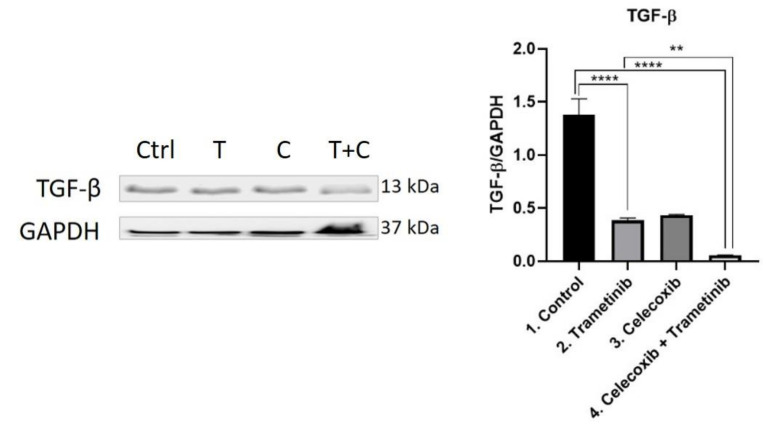
Western blot analysis of TGF-β. Protein expression of TGF-β in BJ + SK-MEL-28 co-culture cells. WB bands were analyzed by densitometry and results were normalized to GAPDH. The left panel illustrates WB bands (Ctrl = control, T = trametinib, C = celecoxib, T + C = trametinib + celecoxib), while right panels illustrate the quantitative analysis of WB results (1 = control, 2 = trametinib, 3 = celecoxib, 4 = celecoxib + trametinib). ** *p* < 0.01, **** *p* < 0.0001. Each bar represents mean ± standard deviation (*n* = 3).

**Stroma remodeling.** TGF-β was evaluated as a key regulator of fibroblasts recruitment and activation at the tumor milieu, via WB (Figure 11). Trametinib and T + C managed to reduce TGF-β expression compared to the control, with a greater inhibitory effect shown in the fourth group (T + C) (mean difference 1.323, 95% CI 1.127 to 1.520, *p* < 0.0001). While the difference between T and C was not significant, the therapeutic combination of T + C significantly reduced TGF-β release compared to trametinib group (mean difference 0.3243, 95% CI 0.1279 to 0.5208, *p* < 0.01).

## 4. Discussion

The antineoplastic effects of celecoxib in vitro are indubitable. Li Gong et al. highlighted that higher doses of celecoxib (IC_50_ in µM range) are needed to exert its antineoplastic effect (such as AKT inhibition), compared with those required to inhibit COX-2 activity (IC_50_ in nM range) [22]. The main problem comes when translating the results in vivo, as the concentrations tested in vitro so far (50µM) are impossible to achieve in human patients. Our work focused on the investigation of the chemopreventive effect of celecoxib (C) in overcoming the activation of PI3K/AKT resistance pathway in metastatic melanoma treated with trametinib (T). Celecoxib (50 nM) and trametinib (25 nM) therapeutic association was tested in vitro on a simulated melanoma tumor formed of SK-MEL-28 human melanoma cells (positive for BRAF V600E and wild type NRAS mutations) and BJ human fibroblasts, used as co-culture. We were particularly interested in the evaluation of the T + C synergism and the impact on proliferation, inflammation, angiogenesis, melanogenesis, therapeutic resistance and tumor–stromal interactions involved in melanoma progression.

Immunotherapy and targeted therapies (dabrafenib and trametinib) remarkably improved progression free survival and overall survival in melanoma patients. However, the response was limited mainly due to resistance mechanisms developed or severe side effects. Kozar et al. described that therapeutic resistance to targeted therapies is mediated by MAPK pathway re-activation, activation of the PI3K/mTOR (mechanistic target of rapamycin) substitutive pathway, stromal cells influence, autophagy and microRNAs (miRNAs). Phenotype switching has an intricate role as well [11]. In up to 80% of cases, MAPK pathway re-activation is a consequence of *NRAS*, *BRAF*, MEK and neurofibromin 1 (*NF1*) alterations [23,24]. Both signal transducer and activator of transcription 3D (STAT3) and MITF transcription factor induce the expression of miR204-5p and miR211-5p, responsible for MAPK or PI3K/AKT pathway re-activation [25,26]. Another strategy that melanoma cells use to adapt to drug-induced stress is by upregulating autophagy, a result of ER stress and TAM receptor protein tyrosine kinases (TYRO3, AXL, MER) pathway activation [27]. Starting from these assumptions, a reasonable way to prevent the development of therapeutic resistance and prolong the benefit would be to personalize standard treatments with chemopreventive agents.

Genetic ablation of COX-2 in *BRAF^V600E^* or *NRAS^G12D^* melanoma models makes them more susceptible to immune control. Moreover, COX-2 inhibition synergizes with anti-PD-1 blockade in promoting the eradication of tumors [28]. For more than 20 years celecoxib has been recommended for its analgesic, anti-inflammatory, and antipyretic effect obtained after inhibition of COX-2 enzyme activity and PGE2 formation via PG G/H synthase-2 downregulation [22]. Recent preclinical and clinical results proved that celecoxib is suitable for cancer therapy and prevention, showing promising results in breast, head and neck, colon and prostate cancer [29,30,31,32]. Celecoxib’s main chemopreventive capabilities that could be used in melanoma include: Inhibition of proliferation and induction of apoptosis through death receptor (caspase-8/caspase-3 activation) and mitochondrial pathways (caspase-9 activation), inhibition of angiogenesis, invasion and prevention of melanoma cell immune escape [33,34,35]. The FDA have already approved celecoxib for increased degradation of *CTNNB1* gene mutations (β-catenin oncoprotein), frequently observed in colon cancer cells, and for reducing the number of colorectal polyps in patients with familial adenomatous polyposis [36,37]. This selective COX-2 inhibitor is able to induce cell cycle arrest at the G2/M phase by increasing p53 levels [38]. Apart from apoptosis, celecoxib also prevented PI3K/AKT-mediated autophagy in SGC-7901 gastric cancer cells [39].

In our study, celecoxib did not impact on cell survival as a single compound. However, viability tests showed that T + C combination induced a time and dose-dependent growth inhibition with the chosen doses. Thus, trametinib and celecoxib potentiated each other’s cytotoxic capabilities in BJ + SK-MEL-28 co-culture. This is consistent with other results reported in the literature, according to which, trametinib sensitized colon cancer cells to celecoxib when they were first tested as a combination [40].

During melanoma staging and as part of the follow-up strategy, the serum LDH is measured as an independent predictive marker for distant metastases and poor prognosis [41,42]. The T + C regimen expressed the highest LDH release compared to the other three groups. This was a direct sign of cell membrane damage and cell death induction as a cumulative inhibitory effect. Comparative FACS analysis following T + C treatment was unanimous with the cytotoxicity and LDH results. The drug combination induced significantly more cell killing via apoptosis (caspase 8 and caspase 3 activation), compared to each treatment used alone. Celecoxib is a potent cytotoxic drug whose antineoplastic effect seems to be COX-2 independent, as it inhibits mitochondrial O_2_ consumption, promotes high reactive oxygen species (ROS) and inhibits antiapoptotic molecules (PDK1 and AKT1) [43,44]. The inhibition of cell proliferation and the induction of enhanced apoptosis was also observed in other melanoma cell lines, such as A375, B16-F10 and Mel Ho [45,46].

Even though NF-kB protein expression was significantly increased in the fourth group compared to the trametinib group, only a small amount was represented by the active form. The result suggests that celecoxib strongly reduced NF-kB activation, explaining the antiproliferative and proapoptotic effect of the therapeutic combination in the SK-MEL-28 + BJ co-culture cells. Moreover, the addition of low-dose celecoxib to trametinib also showed benefits in inhibiting one of the major resistance pathways in melanoma, the AKT pathway. Celecoxib induced stronger AKT pathway inhibition than trametinib, its effect aiding the combination therapy. The group of interest significantly reduced AKT protein levels compared to the control and trametinib groups. The results were also consistent even when an AKT pathway inhibitor was used, as the total amount of protein as well as the active form decreased almost by 50%. Oncogenic PI3K/AKT/IKK/NF-kB pathway activation promotes resistance to combination BRAF and MEK inhibitors [47]. This happens due to multiple genetic mutations, activation of tyrosine kinase receptors (TKRs) or deletion of PTEN [48,49]. Our results suggest that low-dose celecoxib could be used to prevent AKT resistance pathway activation in the dabrafenib and trametinib combination, extending the tumor control. Moreover, melanoma immune evasion results due to COX-2 overexpression and PD-1/PD-L1 interaction. COX-2 can modulate PD-L1 expression through AKT, NF-kB and STAT3 promotion [50,51]. Recent data show that celecoxib down-regulates PD-L1 via FKBP5 in murine glioma stem cells, proving a suitable adjuvant for PD-1 inhibitors as well [12].

COX-2 and pigmentation are closely linked in melanoma. Recent studies highlighted that COX-2-mediated PGE2, like other proinflammatory mediators (interleukin 33, interleukin 18, interferon γ), promotes melanogenesis [52]. The link between inflammatory mediators and alpha-melanocyte stimulating hormone (α-MSH) is stronger than previously thought: After silencing COX-2 in melanocytes, melanin production decreases as a result of MITF and tyrosinase enzyme inhibition [53]. Additionally, MITF is a key melanocyte-specific transcription factor involved in the epithelial to mesenchymal phenotype switching involved in melanoma therapeutic resistance and proliferation. During treatment, when melanoma cells bypass BRAF inhibition, MITF expression inversely correlates with AXL, a TKRs family member [54,55]. Therefore, tyrosinase and MITF protein expression were evaluated. Both pigment markers decreased significantly under the combined regimen, compared to the untreated cells or the ones treated with trametinib. This suggests that celecoxib and trametinib more markedly inhibited tumor-associated melanogenesis and prevented melanoma phenotype switching.

Hypoxia leads to COX-2 and VEGF upregulation within tumors. In order to sustain tumor development, COX-2 also stimulates neoangiogenesis through PI3K/PKC pathway induced VEGF expression [56]. We wanted to further evaluate the anti-angiogenic effect of our association in the proposed melanoma model, as COX-2 inhibitors primarily target angiogenesis. Therefore, HIF-1α and VEGF levels were analyzed and it was observed that trametinib expressed the strongest anti-angiogenic effect. T + C also managed to inhibit HIF-1 and VEGF expression compared to controls. This is consistent with other results, where the anti-angiogenic effect of celecoxib was attributed to PTEN/PI3K/AKT/HIF-1 modulation in a murine hepatocarcinoma model [57].

In order to simulate closer to real melanoma conditions and observe the stromal influence on the therapeutic response, we decided to carry all experiments on human melanoma and fibroblasts cells co-culture. Malignant melanocytes are not the only ones that drive melanoma development. Melanoma has a very heterogenic microenvironment based on altered communication between neoplastic and non-malignant cell populations (fibroblasts, endothelial and inflammatory cells) in the tumor stroma. Cutaneous melanoma begins generally at the dermo-epidermal junction, suggesting an extensive interaction between melanoma cells and dermal fibroblasts [58]. Normal fibroblasts inhibit anarchic cell proliferation, prevent further development of premalignant lesions and form a protective shield against tumor dermal invasion [59]. Along with melanoma progression, TGF-β released at the tumor milieu stimulates the shift of normal fibroblasts into cancer-associated fibroblasts (CAFs). CAFs acquire myofibroblast properties which contribute to diseased tissue remodeling and express markers to sustain cell proliferation and tumor growth: Such as fibroblast specific protein 1 (FSP-1), fibroblast activation protein-alpha (FAP-α), α-smooth muscle actin (α-SMA) and platelet-derived growth factor receptor (PDGFR). [60,61,62]. It has been shown that stromal cells also contribute to BRAF-inhibitor resistance by growth factors secretion, MAPK or PI3K/AKT pathway activation and increased collagen secretion which creates a thicker extracellular matrix as support for cell proliferation [63]. The increased matrix deposition, as well as fibronectin and collagen reorganization seen in melanoma tumors makes them stiffer than normal tissue [64]. This abundant extracellular matrix prevents the homogenous distribution of different antineoplastic drugs and correlates with higher MITF levels. For this reason, high collagen impacts on melanoma proliferative-invasive tumor switch. Besides stromal fibroblasts, melanoma cells are also in charge of collagen deposition in vivo [60]. In the absence of fibroblasts, melanoma differentiation is driven by collagen stiffness via the YAP/PAX3/MITF axis [65]. Such tumors with abundant collagen deposition together with increased MITF target gene expression are associated with poor prognosis [66]. However, in the presence of fibroblasts, TGF-β inhibits PAX3 expression and promotes an invasive phenotype via YAP/TEAD/SMAD transcription [67]. Miskolczi et al. concluded there are two types of melanoma tumors characterized by high collagen production: Ones with high and others with low MITF gene expression, each being inversely correlated with fibroblasts tumor infiltration. The reason for this is that fibroblasts secrete factors that reduce MITF expression [63]. Celecoxib was able to inhibit TGF-β stromal expression in A549 lung cancer cells, preventing cancer cells migration and invasion via SIRT1 downregulation [68]. Our results showed that low-dose celecoxib can also normalize the tumor microenvironment, block the heterogeneous drug distribution and reduce both TGF-β and MITF expression in T + C group [69].

COX-2 overexpression was linked with tumor genesis and progression and proved to be a negative, stage-dependent prognostic factor in melanoma [33]. For this reason, targeting COX-2 using celecoxib as chemoprevention or an adjuvant seemed a logical approach for many preclinical studies, and even for recent clinical trials, in different types of cancer [70,71,72,73]. Preclinical studies have shown that celecoxib and trametinib association increase dabrafenib efficacy and reduce BRAF-inhibitor-induced keratoacanthoma and squamous cell carcinoma by PLX7420 [74,75]. However, the optimal dose should be selected carefully as dabrafenib inhibits CYP450 isoenzymes which are responsible for celecoxib metabolization [76]. Therefore, its co-administration would decrease plasma concentrations as well as the adjuvant efficacy of celecoxib [77]. Another important aspect is that the plasma peak was hard to establish in the case of celecoxib, mainly due to reported associations between genomic disparities of CYP2C9 and plasma drug levels [32]. Despite this, celecoxib increased overall response to chemotherapy. Encouraging results were obtained after the association of celecoxib and paclitaxel in metastatic melanoma patients enrolled in a phase II clinical trial [20]. Yet, different studies appear to contradict each other, raising suspicions on celecoxib’s real clinical benefit and limiting its further investigation. Thus far, the results of a large prospective cohort study concluded that NSAIDs alone are not good candidates for melanoma chemoprevention. However, the study had a few limitations, as the average follow-up was only 5 years and important information in regard to melanoma risk factors and the actual NSAIDs dose/day was missing [78]. In contradiction with these results is a newer study (NCT00000611) that shows postmenopausal Caucasian women who used aspirin, but not other NSAIDs, had a 21% lower melanoma risk. Interestingly, the chemopreventive effect increases with drug duration, with a clear benefit after 5 years [79,80]. Thus, one must weight potential benefits against potential risks.

This in vitro study presents preliminary results that require further confirmation via in vivo experiments for accurate extrapolation to humans. Moreover, deeper understanding of the intricate melanoma biology and celecoxib’s capabilities are needed.

## 5. Conclusions

We all know that in the evolution of real knowledge, contradictions mark the first step in progress towards victory. Our results change the limited beliefs about celecoxib’s use, showing for the first time that 50 nM celecoxib exerts antitumor effects as well. Low-dose celecoxib is still able to increase melanoma cell sensitivity to trametinib and improve the overall treatment response of SK-MEL-28 and BJ co-culture cells in vitro. The beneficial effect of low-dose celecoxib addition to the already used trametinib resides mainly in decreased PI3K/AKT resistance pathway activation, inflammation, melanogenesis and increased apoptosis, enhancing the overall treatment response. There were no reported drug interactions between celecoxib and trametinib so far. Besides, celecoxib and trametinib therapeutic association proved to be more effective than either inhibitor alone. These results definitely encourage further in vivo and clinical research on celecoxib in melanoma. For future research studies, we think that a practical and cost-effective method to evaluate celecoxib’s potential chemopreventive effect in melanoma would involve short-term biomarker modulation studies. These results may predict if low doses of celecoxib can prolong the therapeutic response to targeted or immunotherapies.

## Data Availability

The data presented in this study are openly available at doi…

## Abbreviations

C	celecoxib
T	trametinib
T + C	celecoxib + trametinib
NF-κB	nuclear factor-kappa B
p NF-κB	phosphorylated nuclear factor-kappa B
PI3K	phosphatidylinositol 3-kinase
mTOR	mechanistic target of rapamycin
panAKT	protein kinase B
pan pAKT	phosphorylated protein kinase B
IKK	IkB kinase
MAPK	mitogen activated protein kinase
STAT3	signal transducer and activator of transcription 3
PTEN	phosphatase and tensin homolog
MITF	microphthalmia transcription factor
COX-2	cyclooxygenase-2
PGE2	prostaglandin E2
VEGF	vascular endothelial growth factor
HIF-1α	hypoxia inducible factor–1 alpha
TGF- β	transforming growth factor beta
LDH	lactate dehydrogenase
GAPDH	glyceraldehyde 3-phosphate dehydrogenase
PD-1/PD-L1	programmed ccell death–1/programmed cell death-ligand 1
TAM	TAM receptor protein tyrosine kinases (TYRO3, AXL, MER)
α-MSH	alpha-melanocyte stimulating hormone
CAFs	cancer-associated fibroblasts
FSP-1	fibroblast specific protein 1
FAP-α	fibroblast activation protein-alpha
α-SMA	α-smooth muscle actin
PDGFR	platelet-derived growth factor receptor
YAP/PAX 3	yes-associated protein 65/paired box gene 3
TEAD/SMAD	transcriptional enhancer factor TEF-1 domain family member 1
SIRT1	family of proteins that are the main signal transducers for the receptors of the TGF-β

## References

[1] Roesch A., Melanoma B.C. (2020). Braun-Falco’s Dermatology.

[2] Whiteman D.C., Green A.C., Olsen C.M. (2016). The growing burden of invasive melanoma: Projections of incidence rates and numbers of new cases in six susceptible populations through 2031. J. Investig. Dermatol..

[3] Leonardi G.C., Falzone L., Salemi R., Zanghì A., Spandidos D.A., Mccubrey J.A., Candido S., Libra M. (2018). Cutaneous melanoma: From pathogenesis to therapy. Int. J. Oncol..

[4] Fang S., Xu T., Xiong M., Zhou X., Wang Y., Haydu L.E., Ross M.I., Gershenwald J.E., Prieto V.G., Cormier J.N. (2019). Role of immune response, inflammation and tumor immune response–related cytokines/chemokines in melanoma progression. J. Invest. Dermatol..

[5] Schadendorf D., van Akkooi A.C., Berking C., Griewank K.G., Gutzmer R., Hauschild A., Stang A., Roesch A., Ugurel S. (2018). Melanoma. Lancet.

[6] Al Emran A., Tseng H.Y., Coleman M.C., Tiffen J., Cook S., McGuire H., Gallagher S., Feng C., Hersey P. (2020). Do innate killing mechanisms activated by in-flammasomes have a role in treating melanoma?. Pigm. Cell Melanoma Res..

[7] Lombardo N., Della Corte M., Pelaia C., Piazzetta G., Lobello N., Del Duca E., Bennardo L., Nisticò S.P. (2021). Primary Mucosal Melanoma Presenting with a Unilateral Nasal Obstruction of the Left Inferior Turbinate. Medicina.

[8] Robert C., Grob J.J., Stroyakovskiy D., Karaszewska B., Hauschild A., Levchenko E., Chiarion Sileni V., Schachter J., Garbe C., Bondarenko I. (2019). Five-year outcomes with dabrafenib plus trametinib in metastatic melanoma. N. Engl. J. Med..

[9] Ribas A., Wolchok J.D. (2018). Cancer immunotherapy using checkpoint blockade. Science.

[10] Sharma P., Hu-Lieskovan S., Wargo J.A., Ribas A. (2017). Primary, adaptive, and acquired resistance to cancer immunotherapy. Cell.

[11] Kozar I., Margue C., Rothengatter S., Haan C., Kreis S. (2019). Many ways to resistance: How melanoma cells evade targeted therapies. Biochim. Biophys. Acta Rev. Cancer.

[12] Hayes T.K., Luo F., Cohen O., Goodale A.B., Lee Y., Pantel S., Bagul M., Piccioni F., Root D.E., Garraway L.A. (2019). A Functional Landscape of Resistance to MEK1/2 and CDK4/6 Inhibition in NRAS-Mutant Melanoma. Cancer Res..

[13] Arozarena I., Wellbrock C. (2019). Phenotype plasticity as enabler of melanoma progression and therapy resistance. Nat. Rev. Cancer.

[14] Varedi A., Rahman H., Kumar D., Catrow J.L., Cox J.E., Liu T., Florell S.R., Boucher K.M., Okwundu N., Burnett W.J. (2020). ASA Suppresses PGE2 in Plasma and Melanocytic Nevi of Human Subjects at Increased Risk for Melanoma. Pharmaceut.

[15] Minisini A.M., Pascoletti G., Intersimone D., Poletto E., Driol P., Spizzo R., Scott C.A., Puglisi F., Fasola G., Di Loreto C. (2013). Expression of thymidine phosphorylase and cyclooxygenase-2 in melanoma. Melanoma Res..

[16] Becker M.R., Siegelin M.D., Rompel R., Enk A.H., Gaiser T. (2009). COX-2 expression in malignant melanoma: A novel prognostic marker?. Melanoma Res..

[17] Ferreira M., Krykbaeva I., Damsky W., Kluger H.M., Bosenberg M. (2019). Evaluating the role of the COX2/PGE2 pathway in anti-melanoma immunity. Int. J. Clin. Oncol..

[18] Mao Y., Poschke I., Wennerberg E., De Coaña Y.P., Brage S.E., Schultz I., Hansson J., Masucci G., Lundqvist A., Kiessling R. (2013). Melanoma-educated CD14+ cells acquire a mye-loid-derived suppressor cell phenotype through COX-2–dependent mechanisms. Cancer Res..

[19] Goulet A.C., Einsphar J.G., Alberts D.S., Beas A., Burk C., Bhattacharyya A.K., Bangert J., Harmon J.M., Fujiwara H., Koki A. (2003). Analysis of cyclooxygenase 2 (COX-2) ex-pression during malignant melanoma progression. Cancer Biol Ther..

[20] Bhatt R.S., Merchan J., Parker R., Wu H.K., Zhang L., Seery V., Heymach J.V., Atkins M.B., McDermott D., Sukhatme V.P. (2010). A phase 2 pilot trial of low-dose, continuous infusion, or “metronomic” paclitaxel and oral celecoxib in patients with metastatic melanoma. Cancer Interdiscip. Int. J. Am. Cancer Soc..

[21] Yamaguchi I., Nakajima K., Shono K., Mizobuchi Y., Fujihara T., Shikata E., Yamaguchi T., Kitazato K., Sampetrean O., Saya H. (2020). Downregulation of PD-L1 via FKBP5 by celecoxib augments antitumor effects of PD-1 blockade in a malignant glioma model. Neuro-Oncol. Adv..

[22] Gong L., Thorn C.F., Bertagnolli M.M., Grosser T., Altman R.B., Klein T.E. (2012). Celecoxib pathways: Pharmacokinetics and pharma-codynamics. Pharm. Genom..

[23] Dietrich P., Kuphal S., Spruss T., Hellerbrand C., Bosserhoff A.K. (2018). Wild-type KRAS is a novel therapeutic target for melanoma contributing to primary and acquired resistance to BRAF inhibition. Oncogene.

[24] Stark M.S., Bonazzi V.F., Boyle G.M., Palmer J.M., Symmons J., Lanagan C.M., Schmidt C.W., Herington A.C., Ballotti R., Pollock P.M. (2015). miR-514a regulates the tumour suppressor NF1 and modulates BRAFi sensitivity in melanoma. Oncotarget.

[25] Fattore L., Costantini S., Malpicci D., Ruggiero C.F., Ascierto P.A., Croce C.M., Mancini R., Ciliberto G. (2017). MicroRNAs in melanoma development and resistance to target therapy. Oncotarget.

[26] Kozar I., Cesi G., Margue C., Philippidou D., Kreis S. (2017). Impact of BRAF kinase inhibitors on the miRNomes and transcriptomes of melanoma cells. Biochim. Biophys. Acta Gen. Subj..

[27] Liu X., Wu J., Qin H., Xu J. (2018). The role of autophagy in the resistance to BRAF inhibition in BRAF-Mutated melanoma. Target. Oncol..

[28] Zelenay S., Van Der Veen A.G., Böttcher J.P., Snelgrove K.J., Rogers N., Acton S.E., Chakravarty P., Girotti M.R., Marais R., Quezada S.A. (2015). Cyclooxygenase-dependent tumor growth through evasion of immunity. Cell.

[29] Saji S., Hirose M., Toi M. (2004). Novel sensitizing agents: Potential contribution of COX-2 inhibitor for endocrine therapy of breast cancer. Breast Cancer.

[30] Harris R.E. (2009). Cyclooxygenase-2 (cox-2) blockade in the chemoprevention of cancers of the colon, breast, prostate, and lung. Inflammopharmacology.

[31] Patil V.M., Noronha V., Joshi A., Abhyankar A., Menon N., Dhumal S., Prabhash K. (2020). Beyond conventional chemotherapy, tar-geted therapy and immunotherapy in squamous cell cancer of the oral cavity. Oral. Oncol..

[32] Hedberg M.L., Peyser N.D., Bauman J.E., Gooding W.E., Li H., Bhola N.E., Zhu T.R., Zeng Y., Brand T.M., Kim M.O. (2019). Use of nonsteroidal anti-inflammatory drugs predicts improved patient survival for PIK3CA-altered head and neck cancer. J. Exp. Med..

[33] Tudor D.V., Bâldea I., Lupu M., Kacso T., Kutasi E., Hopârtean A., Stretea R., Filip A.G. (2020). COX-2 as a potential biomarker and therapeutic target in melanoma. Cancer Biol. Med..

[34] Kim N., Kim C.H., Ahn D.W., Lee K.S., Cho S.J., Park J.H., Lee M.K., Kim J.S., Jung H.C., Song I.S. (2009). Anti-gastric cancer effects of celecoxib, a selective COX-2 inhibitor, through inhibition of Akt signaling. J. Gastroenterol. Hepatol..

[35] Rosas C., Sinning M., Ferreira A., Fuenzalida M., Lemus D. (2014). Celecoxib decreases growth and angiogenesis and promotes apoptosis in a tumor cell line resistant to chemotherapy. Biol. Res..

[36] Patel J.M., Knopf J., Reiner E., Bossuyt V., Epstein L., DiGiovanna M., Chung G., Silber A., Sanft T., Hofstatter E. (2016). Mutation based treatment recommendations from next generation sequencing data: A comparison of web tools. Oncotarget.

[37] Maier T.J., Janssen A., Schmidt R., Geisslinger G., Grösch S. (2005). Targeting the beta-catenin/APC pathway: A novel mechanism to explain the cyclooxygenase-2-independent anticarcinogenic effects of celecoxib in human colon carcinoma cells. FASEB J..

[38] Setiawati A., Setiawati A. (2016). Celecoxib, a COX-2 Selective Inhibitor, Induces Cell Cycle Arrest at the G2/M Phase in HeLa Cer-vical Cancer Cells. Asian Pac. J. Cancer Prev..

[39] Liu M., Li C.M., Chen Z.F., Ji R., Guo Q.H., Li Q., Zhang H.L., Zhou Y.N. (2014). Celecoxib regulates apoptosis and autophagy via the PI3K/Akt signaling pathway in SGC-7901 gastric cancer cells. Int. J. Molec. Med..

[40] Pauli C., Hopkins B.D., Prandi D., Shaw R., Fedrizzi T., Sboner A., Sailer V., Augello M., Puca L., Rosati R. (2017). Personalized in vitro and in vivo cancer models to guide precision medicine. Cancer Discov..

[41] Garbe C., Amaral T., Peris K., Hauschild A., Arenberger P., Bastholt L., Bataille V., Del Marmol V., Dréno B., Fargnoli M.C. (2020). European consensus-based interdisciplinary guideline for melanoma. Part 1: Diagnostics–Update 2019. Eur. J. Cancer..

[42] Agarwala S.S., Keilholz U., Gilles E., Bedikian A.Y., Wu J., Kay R., Stein C.A., Itri L.M., Suciu S., Eggermont A.M. (2009). LDH correlation with survival in advanced melanoma from two large, randomised trials (Oblimersen GM301 and EORTC 18951). Eur. J. Cancer..

[43] Pritchard R., Rodríguez-Enríquez S., Pacheco-Velázquez S.C., Bortnik V., Moreno-Sánchez R., Ralph S. (2018). Celecoxib inhibits mi-tochondrial O2 consumption, promoting ROS dependent death of murine and human metastatic cancer cells via the apop-totic signalling pathway. Biochem. Pharmacol..

[44] Jendrossek V., Handrick R., Belka C. (2003). Celecoxib activates a novel mitochondrial apoptosis signaling pathway. FASEB J..

[45] Bundscherer A., Hafner C., Maisch T., Becker B., Landthaler M., Vogt T. (2008). Antiproliferative and proapoptotic effects of rapamycin and celecoxib in malignant melanoma cell lines. Oncol. Rep..

[46] Sadhu S.S., Wang S., Averineni R.K., Seefeldt T., Yang Y., Guan X. (2016). In-vitro and in-vivo inhibition of melanoma growth and me-tastasis by the drug combination of celecoxib and dacarbazine. Melanoma Res..

[47] Irvine M., Stewart A., Pedersen B., Boyd S., Kefford R., Rizos H. (2018). Oncogenic PI3K/AKT promotes the step-wise evolution of combination BRAF/MEK inhibitor resistance in melanoma. Oncogenesis.

[48] Zuo Q., Liu J., Huang L., Qin Y., Hawley T., Seo C., Merlino G., Yu Y. (2018). AXL/AKT axis mediated-resistance to BRAF inhibitor depends on PTEN status in melanoma. Oncogene.

[49] Miller M.A., Oudin M.J., Sullivan R.J., Wang S.J., Meyer A.S., Im H., Frederick D.T., Tadros J., Griffith L.G., Lee H. (2016). Reduced proteolytic shedding of receptor tyrosine kinases is a post-translational mechanism of kinase inhibitor resistance. Cancer Discov..

[50] Botti G., Fratangelo F., Cerrone M., Liguori G., Cantile M., Anniciello A.M., Scala S., D’Alterio C., Trimarco C., Ianaro A. (2017). COX-2 expression positively correlates with PD-L1 expression in human melanoma cells. J. Trans. Med..

[51] Prima V., Kaliberova L.N., Kaliberov S., Curiel D.T., Kusmartsev S. (2017). COX2/mPGES1/PGE2 pathway regulates PD-L1 expression in tumor-associated macrophages and myeloid-derived suppressor cells. Proc. Natl. Acad. Sci. USA.

[52] Fu C., Chen J., Lu J., Yi L., Tong X., Kang L., Pei S., Ouyang Y., Jiang L., Ding Y. (2020). Roles of inflammation factors in melanogenesis. Mol. Med. Rep..

[53] Kim J.Y., Shin J.Y., Kim M.R., Hann S.K., Oh S.H. (2012). siRNA-mediated knock-down of COX-2 in melanocytes suppresses melano-genesis. Exp Dermatol..

[54] Tirosh I., Izar B., Prakadan S.M., Wadsworth M.H., Treacy D., Trombetta J.J., Rotem A., Rodman C., Lian C., Murphy G. (2016). Dissecting the multicellular ecosystem of metastatic melanoma by single-cell RNA-seq. Science.

[55] Smith M.P., Rana S., Ferguson J., Rowling E.J., Flaherty K.T., Wargo J.A., Marais R., Wellbrock C. (2019). A PAX3/BRN2 rheostat controls the dynamics of BRAF mediated MITF regulation in MITFhigh/AXLlow melanoma. Pigment Cell Melanoma Res..

[56] Fosslien E. (2001). Molecular pathology of cyclooxygenase-2 in cancer-induced angiogenesis. Ann. Clin. Lab. Sci..

[57] Sui W., Zhang Y., Wang Z., Wang Z., Jia Q., Wu L., Zhang W. (2014). Antitumor effect of a selective COX-2 inhibitor, celecoxib, may be attributed to angiogenesis inhibition through modulating the PTEN/PI3K/Akt/HIF-1 pathway in an H22 murine hepatocar-cinoma model. Oncol. Rep..

[58] Cirri P., Chiarugi P. (2011). Cancer associated fibroblasts: The dark side of the coin. Am. J. Cancer Res..

[59] Bissell M.J., Hines W.C. (2011). Why don’t we get more cancer? A proposed role of the microenvironment in restraining cancer pro-gression. Nat. Med..

[60] Zhou L., Yang K., Andl T., Wickett R.R., Zhang Y. (2015). Perspective of targeting cancer-associated fibroblasts in melanoma. J. Cancer..

[61] Augsten M. (2014). Cancer-associated fibroblasts as another polarized cell type of the tumor microenvironment. Front. Oncol..

[62] Räsänen K., Vaheri A. (2010). Activation of fibroblasts in cancer stroma. Exp. Cell Res..

[63] Miskolczi Z., Smith M.P., Rowling E.J., Ferguson J., Barriuso J., Wellbrock C. (2018). Collagen abundance controls melanoma phenotypes through lineage-specific microenvironment sensing. Oncogene.

[64] Kirkpatrick S.J., Wang R.K., Duncan D.D., Kulesz-Martin M., Lee K. (2006). Imaging the mechanical stiffness of skin lesions by in vivo acousto-optical elastography. Opt. Express.

[65] Smith M.P., Brunton H., Rowling E.J., Ferguson J., Arozarena I., Miskolczi Z., Lee J.L., Girotti M.R., Marais R., Levesque M.P. (2016). Inhibiting drivers of non-mutational drug tolerance is a salvage strategy for targeted melanoma therapy. Cancer Cell.

[66] Wellbrock C., Arozarena I. (2015). Microphthalmia-associated transcription factor in melanoma development and MAP-kinase pathway targeted therapy. Pigment Cell Melanoma Res..

[67] Yang G., Li Y., Nishimura E.K., Xin H., Zhou A., Guo Y., Dong L., Denning M.F., Nickoloff B.J., Cui R. (2008). Inhibition of PAX3 by TGF-beta modulates melanocyte viability. Mol. Cell.

[68] Cha B.K., Kim Y.S., Hwang K.E., Cho K.H., Oh S.H., Kim B.R., Jun H.Y., Yoon K.H., Jeong E.T., Kim H.R. (2016). Celecoxib and sulindac inhibit TGF-β1-induced epitheli-al-mesenchymal transition and suppress lung cancer migration and invasion via downregulation of sirtuin 1. Oncotarget.

[69] Zhang B., Jin K., Jiang T., Wang L., Shen S., Luo Z., Tuo Y., Liu X., Hu Y., Pang Z. (2017). Celecoxib normalizes the tumor microenvironment and enhances small nanotherapeutics delivery to A549 tumors in nude mice. Sci. Rep..

[70] ClinicalTrials.gov Schrump (MD): National Institutes of Health Clinical Center (US). Identifier: NCT01341496, Epigenetically-Modified Autologous Tumor Cell Vaccs and ISCOMATRIX(TM) Adjuvant with Metronomic Oral Cyclophosphamide and Celecoxib in Pts Undergoing Resection of Sarcomas, Melanomas, Germ Cell Tumors, or Epithelial Malignancies Metastatic to Lungs, Pleura or Mediastinum. NCT01341496.

[71] Tołoczko-Iwaniuk N., Dziemiańczyk-Pakieła D., Nowaszewska B.K., Celińska-Janowicz K., Miltyk W. (2019). Celecoxib in Cancer Therapy and Prevention–Review. Curr. Drug Targets.

[72] European Celecoxib Trial in Primary Breast Cancer—Clinical Trials.gov. https://clinicaltrials.gov/ct2/show/NCT02429427?term=celecoxib&cond=cancer&draw=2&rank=1.

[73] Celecoxib in Preventing Non-Small Cell Lung Cancer in Tobacco Smokers—Clinical Trials.gov. https://clinicaltrials.gov/ct2/show/NCT00020878?term=celecoxib&cond=cancer&draw=2&rank=4.

[74] Escuin-Ordinas H., Atefi M., Fu Y., Cass A., Ng C., Huang R.R., Yashar S., Comin-Anduix B., Avramis E., Cochran A.J. (2014). COX-2 inhibition prevents the appearance of cutaneous squamous cell carcinomas accelerated by BRAF inhibitors. Mol. Oncol..

[75] Paton E.L., Turner J.A., Schlaepfer I.R. (2020). Overcoming Resistance to Therapies Targeting the MAPK Pathway in BRAF-Mutated Tumours. J Oncol..

[76] Wang B., Wang J., Huang S.Q., Su H.H., Zhou S.F. (2009). Genetic polymorphism of the human cytochrome P450 2C9 gene and its clinical significance. Curr. Drug Metab..

[77] Lawrence S.K., Nguyen D., Bowen C., Richards-Peterson L., Skordos K.W. (2014). The metabolic drug-drug interaction profile of Dabrafenib: In vitro investigations and quantitative extrapolation of the P450-mediated DDI risk. Drug Metab. Dispos..

[78] Asgari M.M., Maruti S.S., White E. (2008). A large cohort study of nonsteroidal anti-inflammatory drug use and melanoma incidence. JNCI J. Natl. Cancer Inst..

[79] Gamba C.A., Swetter S.M., Stefanick M.L., Kubo J., Desai M., Spaunhurst K.M., Sinha A.A., Asgari M.M., Sturgeon S., Tang J.Y. (2013). Aspirin is associated with lower melanoma risk among postmenopausal Caucasian women: The Women’s Health Initiative. Cancer.

[80] Goodman J.R., Grossman D. (2014). Aspirin and other NSAIDs as chemoprevention agents in melanoma. Cancer Prev. Res..

